# The dynamics of patient visits to traditional Chinese medicine during the 2019 coronavirus pandemic

**DOI:** 10.1186/s12906-021-03245-x

**Published:** 2021-02-19

**Authors:** Shun-Ku Lin, Chien-Tung Wu, Hui-Jer Chou, Chia-Jen Liu, Fu-Yang Ko, Ching-Hsuan Huang, Jung-Nien Lai

**Affiliations:** 1 Institute of Public Health, National Yang-Ming University, Taipei, Taiwan, Republic of China; 2 Department of Chinese Medicine, Taipei City Hospital Renai Branch, Taipei, Taiwan, Republic of China; 3 University of Taipei, Taipei, Taiwan, Republic of China; 4 Institute of Traditional Medicine, School of Medicine, National Yang-Ming University, Taipei, Taiwan, Republic of China; 5 Department of Chinese Medicine, Taipei City Hospital Linsen Chinese Medicine and Kunming Branch, Taipei, Taiwan, Republic of China; 6 School of Medicine, National Yang-Ming University, Taipei, Taiwan, Republic of China; 7 Division of Hematology and Oncology, Department of Medicine, Taipei Veterans General Hospital, Taipei, Taiwan, Republic of China; 8 National Union of Chinese Medical Doctor’s Association, Taipei, Taiwan, Republic of China; 9 Department of Chinese Traumatology Medicine, China Medical University Hospital, Taichung, Taiwan, Republic of China; 10 School of Chinese Medicine, College of Chinese Medicine, China Medical University, No.91, Hsueh-Shih Road, Taichung, 40402 Taiwan, Republic of China; 11 Graduate Institute of Integrated Medicine, China Medical University, Taichung, Taiwan, Republic of China; 12 Department of Chinese Medicine, China Medical University Hospital, Taichung, Taiwan, Republic of China

**Keywords:** COVID-19 pandemic, Traditional Chinese medicine, Utilization, Mobile medicine

## Abstract

**Background:**

Large-scale epidemics have changed people’s medical behavior, and patients tend to delay non-urgent medical needs. However, the impact of the pandemic on the use of complementary and alternative medicine remains unknown.

**Methods:**

This retrospective study aimed to analyze the changes in the number of traditional Chinese medicine (TCM) patients and examine the epidemic prevention policy during the coronavirus disease 2019 (COVID-19) pandemic. We analyzed the number of TCM patients in Taipei City Hospital from January 2017 to May 2020. We tallied the numbers of patients in each month and compared them with those in the same months last year. We calculated the percentage difference in the number of patients to reveal the impact of the COVID-19 pandemic on TCM utilization. We used the Mann–Whitney U test to examine whether there was a significant difference in the number of patients during the COVID-19 pandemic.

**Results:**

We included a total of 1,935,827 TCM visits of patients from January 2017 to May 2020 in this study. During the COVID-19 pandemic, the number of patients decreased significantly, except in February 2020. The number of patients during the COVID-19 pandemic had fallen by more than 15% compared with those in the same months last year. March and April had the greatest number of patient losses, with falls of 32.8 and 40% respectively. TCM patients declined significantly during the COVID-19 pandemic, and mobile medicine provided to rural areas fell considerably. Among all the TCM specialties, pediatrics and traumatology, as well as infertility treatment, witnessed the most significant decline in the number of patients. However, the number of cancer patients has reportedly increased.

**Conclusions:**

The COVID-19 pandemic decreased the utilization rate of TCM, especially for mobile healthcare in rural areas. We suggest that the government pay attention to the medical disparity between urban and rural areas, which are affected by the pandemic, as well as allocate adequate resources in areas deprived of medical care.

## Background

Large-scale epidemics have changed the people’s medical behavior and medical utilization [1].

During the Severe Acute Respiratory Syndrome (SARS) epidemic in 2003, Taiwan’s medical utilization rate and medical expenses dropped remarkably [2]. Patients tended to delay non-urgent medical needs and postpone elective procedures. However, the use of traditional Chinese medicine (TCM) during SARS marginally increased (1.8%) in Taiwan. This could be attributed to people choosing complementary and alternative medicine as a substitute for Western medicine [3]. The mode of transmission of coronavirus disease 2019 (COVID-19) in Taiwan is different from that of SARS. COVID-19 was transmitted from abroad with limited community transmission, while SARS was mainly transmitted via nosocomial infection. The impact of the current pandemic on the use of complementary and alternative medicine remains unknown. This study aimed to analyze the changes in the number of TCM patients.

## Methods

This retrospective survey aimed to analyze the difference in the number of TCM patients. The Taipei City Hospital’s Research Ethics Committee reviewed and approved this study (review number TCHIRB-10904002-E). We evaluated the number of TCM patient visits in Taipei City Hospital from January 2017 to May 2020, including outpatients and ward consultations. We adopted the method of judging sampling to include all patient visits to the Department of Traditional Chinese Medicine of Taipei City Hospital during this period. Taipei City Hospital consists of ten hospitals and is the largest public hospital in Northern Taiwan. Medical services cater to the entire city, spanning 271.8 km^2^ with 2.6 million residents. The hospital provides TCM services, including outpatient services and ward consultations. It also offers specialized medical services in TCM such as internal medicine, acupuncture, pediatrics, obstetrics and gynecology, and traumatology. Moreover, Taipei City Hospital also provides mobile medical services for rural areas that lack medical resources. Their TCM medical team visits rural clinics weekly. Since Taipei City Hospital has a complete medical system and serves all people in Taipei City, it is suitable as a representative sample of the evaluation of medical utilization [4].

We obtained the number of TCM patients from the hospital information system, including all departments. We computed the number of patients each month and compared it with the same months last year. We divided the patients by the following specialties: internal medicine, acupuncture, traumatology, obstetrics and gynecology, and pediatrics. Furthermore, we separately analyzed the number of stroke, cancer, and infertility patients. Taiwan Health Insurance encourages TCM practitioners to provide medical services for patients with the aforementioned diseases and to increase medical claims. We also scrutinized the number of mobile medical visits in rural areas, including partial townships and outlying islands.

We calculated the percentage difference in the number of patients to reveal the impact of the COVID-19 pandemic on TCM usage. We used three control groups as a benchmark to understand the impact of the pandemic on the number of TCM patients. First, we used the number of patients in Taipei City Hospital in the same months of the previous year (January to May 2019) to compare to the number of patients during the COVID-19 pandemic (January to May 2020). Second, we used the Mann–Whitney U test to examine whether there was a significant disparity between the patients’ visits during the COVID-19 pandemic and that in the same months in the past 3 years (January to May 2017 to 2019). The commonly used statistical method to test two sets of continuous variables is the two-sample t-test. Because we included only five COVID-19 patient visit data (January to May 2017), and it is difficult to judge that the variable population is a normal distribution. Therefore, we adopt the nonparametric statistics method Mann-Whitney U test to avoid false statistical significance. Third, we used the variation in the number of TCM patients nationwide during the SARS epidemic as the control group (from March 2003 to July 2003, using a two million sample dataset of Taiwan’s National Health Insurance Research Database) to compare with the number of patients during COVID-19, then we had the difference between these two numbers. We screened TCM users from Taiwan’s National Health Insurance Research Database and calculated the number of TCM doctors in the country and the percentage difference. We used MedCalc Statistical Software Version 19.4.0 (MedCalc Software Ltd., Ostend, Belgium) to analyze the data.

## Results

**Fig. 1:**
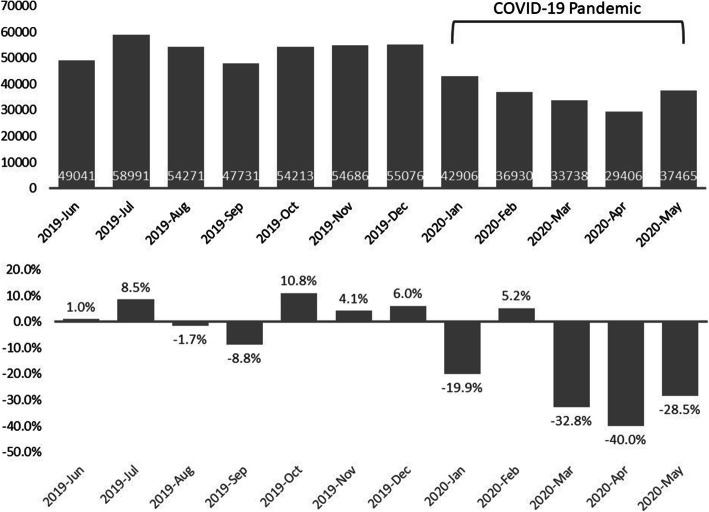
Changes in the number of TCM patients during COVID-19 pandemic. We recorded the number of TCM patients in Taipei City Hospital from June 2019 to May 2020, and the percentage difference in comparison to that in the same months of the previous year. Since the COVID-19 pandemic began to spread worldwide in January 2020, the number of Chinese medicine clinics has dropped significantly, and it has fallen by more than 15% compared to that in the same months last year

The study included a total of 1,935,827 patients who are recipients of the services of Taipei City Hospital. Figure 1 shows the number of patients and the percentage change during the COVID-19 pandemic. The number of patients declined significantly in the present year, except in February 2020. The number of patients has fallen by more than 15% compared to last year. March and April had the substantial number of losses, falling by 32.8 and 40%, respectively.

**Fig. 2:**
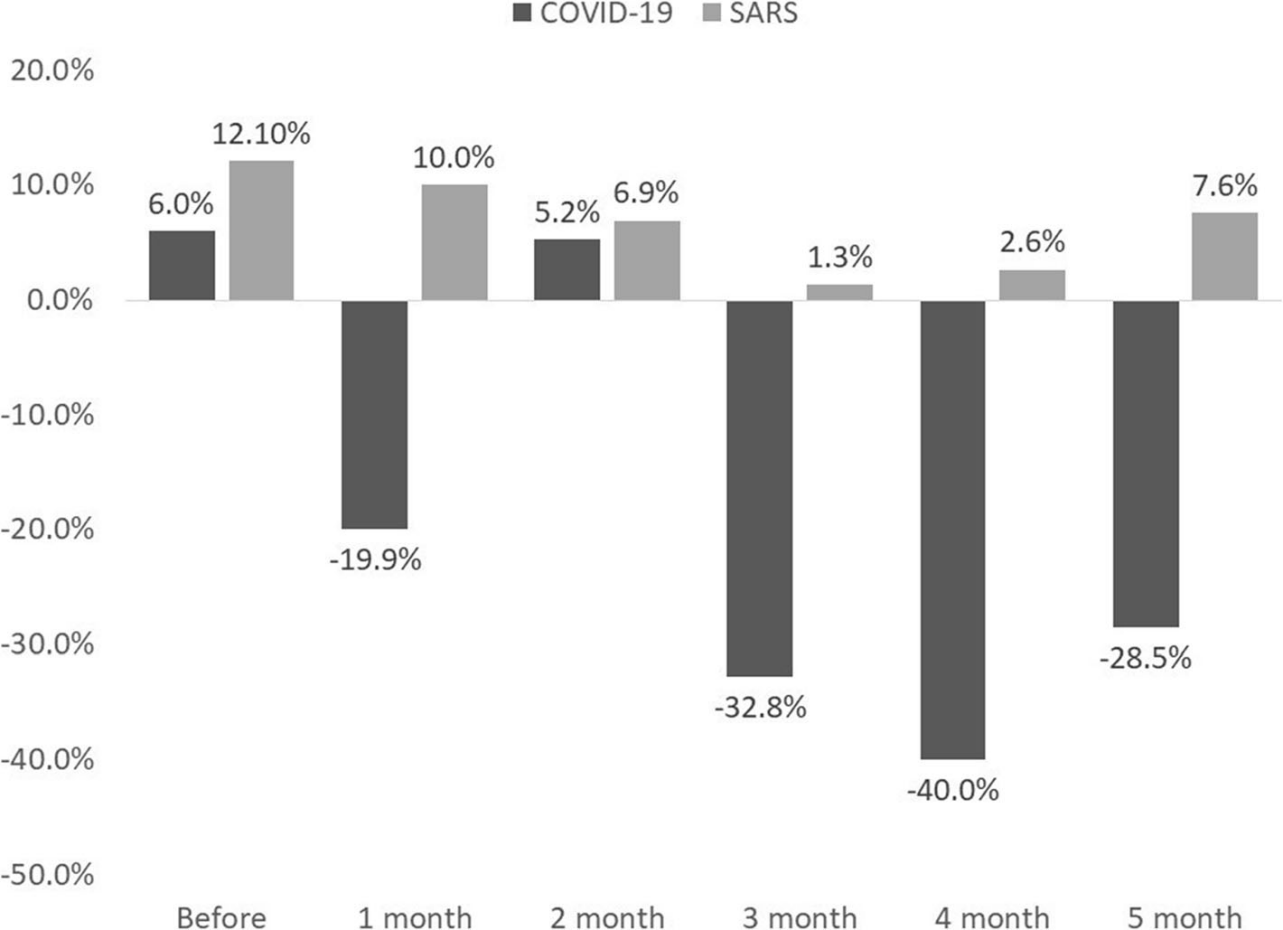
A comparison of the number of TCM patients during the SARS epidemic and the COVID-19 pandemic. The number of TCM patients increased marginally during the SARS epidemic. While, the number of patients utilizing TCM dropped significantly during COVID-19

We compared the number of TCM patients during the SARS epidemic and the COVID-19 pandemic in Fig. 2. We used the main epidemic period of the disease in Taiwan as the comparison standard. The epidemic period of SARS ran from February to July 2003 in Taiwan, and COVID-19 was mainly from January to May 2020. The number of TCM patients increased marginally during the SARS epidemic. On the contrary, the number of patients with TCM dropped significantly during COVID-19.

**Fig. 3:**
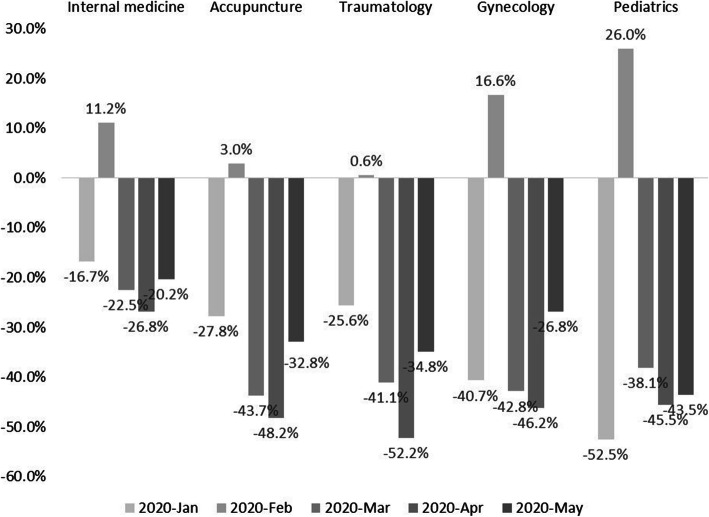
The percentage difference in the number of patients in each specialty in comparison to that in the same months last year. Pediatrics and traumatology received the most significant declines, with the most reductions exceeding 50%. Acupuncture and obstetrics and gynecology also fell by more than 40%, compared to the same months last year

We separately analyzed the number of patients in different TCM specialties and presented the percentage change, also we compared this number to the previous year in Fig. 3. Pediatrics and traumatology received the most significant declines, with the greatest reductions exceeding 50%. Acupuncture and obstetrics and gynecology also fell by more than 40%, compared to the same months last year. Internal medicine had a relatively stable number of patients, with a decrease of only 26.8% at most.

**Fig. 4:**
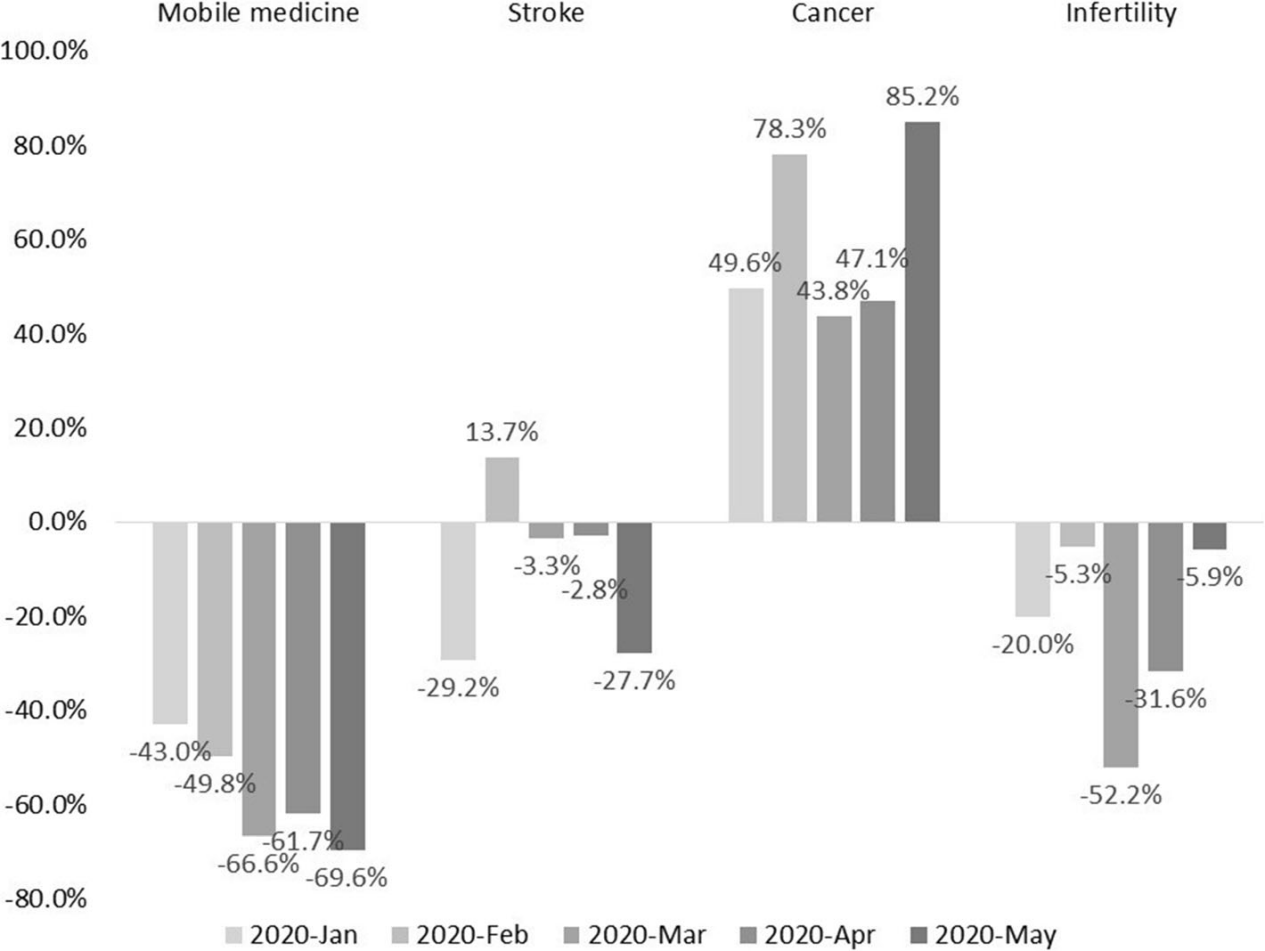
The percentage difference in the number of patients in different TCM treatment programs. During the COVID-19 pandemic, mobile TCM services and infertility treatments in rural areas declined significantly. On the contrary, the number of cancer patients receiving adjuvant therapy with TCM increased significantly as compared with that in the same months last year

Figure 4 shows the percentage change in the number of patients in different TCM treatment programs. The number of infertility treatments declined during the COVID-19 pandemic. As for the sum of cancer patients, on the contrary, it revealed an upward trend. The number of stroke patients treated with TCM has also decreased, but it was not evident as compared to other diseases. Moreover, the number of patients utilizing mobile medicine in rural areas has fallen sharply during the pandemic, falling by more than 60% from March to May 2020.

We used the Mann–Whitney U test to examine whether the patients’ visits declined significantly during the COVID-19 pandemic comparing with the last 3 years (2017 to 2019), as showed in Table 1. The results show that in the departments of internal medicine, acupuncture, traumatology, obstetrics and gynecology, pediatrics, and mobile medicine, the number of medical visits during the COVID-19 pandemic was significantly smaller than that of the same month in the past 3 years, with a *p*-value of less than 0.05. On the other hand, there was no significant difference in the number of patients with specific diseases such as stroke and cancer.

**Table 1:** Significant changes in the number of patients of TCM before and during the COVID-19 pandemic using the Mann–Whitney U test

	Median (IQR) of patient visits before COVID-19 pandemic	Median (IQR) of patient visits after COVID-19 pandemic	Average rank of patient visits before COVID-19 pandemic	Average rank of patient visits after COVID-19 pandemic	Mann–Whitney U	Test statistic Z	Two-tailed probability
Total	50,932(5317)	36,930(6170)	23.306	4.4	7	3.309	0.0009*
Internal medicine	9431(1194)	7512(1023)	23.250	4.8	9	3.227	0.0013*
Acupuncture	5606(1243)	3821(978)	23.120	5.0	11	3.118	0.0018*
Traumatology	2420(263)	1657(391)	23.167	5.4	12	3.108	0.0019*
Obstetrics and Gynecology	1502(218)	927(182)	23.306	4.4	7	3.307	0.0009*
Pediatrics	247(63)	156(50)	23.361	4.0	5	3.387	0.0007*
Mobile medicine	459(87)	155(42)	23.500	3.0	0	3.586	0.0003*
Stroke	969(295)	804(128)	22.250	12.0	45	1.793	0.0730
Cancer	188(76)	228(45)	19.806	29.6	47	−1.714	0.0866

## Discussion

This study revealed that the quantity of TCM patients declined significantly during the COVID-19 pandemic, and mobile medicine provided to rural areas dropped even further. Patients availing TCM specialty services have also decreased, with pediatrics and traumatology having the most significant decline, as well as infertility treatment. However, the number of cancer patients has notably increased.

There was a significant difference in the utilization rate of TCM treatments among Taiwanese patients during SARS and the COVID-19 pandemic. During the SARS epidemic in 2003, the utilization of TCM services increased [3]. Conversely, during the COVID-19 pandemic, the utilization rate of TCM decreased markedly. We surmised that the Taiwanese government used different strategy during SARS in 2003 and used the national network information system and health system to combat the COVID-19 pandemic [5]. During SARS, the spread of the epidemic is concentrated in large hospitals. Therefore, people might consider to receive TCM treatment in local clinic [6, 7]. Some studies have also shown that taking traditional Chinese medicine may help reduce the spread of SARS. However, there are still few papers on the changes in TCM usage during SARS or COVID-19.

The SARS epidemic had spread to more than 30 countries, affecting 8096 people, resulting in 774 deaths worldwide from November 1, 2002 to July 31, 2003. Using the approach during the SARS outbreak, in January, the Taiwanese government issued the COVID-19 case definition and notification process, requiring all physicians (including TCM) to report suspected cases during the outset of COVID-19 in Wuhan. Due to the enormous losses to society because of SARS and considering that COVID-19 can be asymptomatic and is highly infectious, the Taiwanese government implemented stricter restrictions during this pandemic. The subsequent epidemic policy for COVID-19 includes maintaining social distance, wearing face masks in public, and prohibition of gatherings of more than 50 people in a meeting or conference. Therefore, the utilization rate of TCM dropped by more than 30% after March 2020. It is common knowledge that the COVID-19 has spread to more than 188 countries, affecting 14.03 million people, resulting in 602,000 deaths worldwide within only 6 months. Fortunately, compared to the same period in 2003, the proportion of probable SARS cases dropped from 8.2% (664 Taiwanese cases per 8096 global cases) to 0.0032% (766 Taiwanese cases per 77.6 million global cases) in 2020 in Taiwan.

The number of people quarantined at home was 10,904 in the 2003 SARS epidemic and 77,948 in the 2020 COVID-19 pandemic. During COVID-19, the number of people quarantined at home exceeds three-thousandths of the total population, and they are required to seek medical treatment only at designated medical centers. The decline in Chinese medicine utilization can be attributed to the stringent health policies especially for people in quarantine who are only allowed access to medical care at designated health facilities.

Unlike other months, the utilization rate of Chinese medicine in February 2020 has increased slightly (5.2%), compared to February 2019. One possible explanation is that the date of the Chinese New Year affects TCM utilization. The Chinese New Year in Taiwan accompanied a one-week holiday, during which medical institutions will reduce outpatient services. The Chinese New Year holiday in 2020 is in January, and the holiday in 2019 is in February. Therefore, the number of outpatients in Chinese medicine has increased relatively.

Notably, we found that the number of mobile medical services provided to rural areas dropped significantly. Rural areas lack medical resources, and people’s health status is worse than that in urban areas. Taiwan’s Ministry of Health and Welfare started TCM mobile medical services in rural areas in 2004 and provides home medical care for bedridden patients.

However, the present study postulates that a large-scale epidemic of infectious diseases will consume a large amount of medical resources in urban areas and reduce medical accessibility in rural areas. We suggested that the government allocate adequate funds and provide medical workforce to maintain access to medical services and institute fairness in terms of resource allocation to rural areas, especially during the pandemic.

The number of TCM visits from cancer patients increased from 43.8 to 85.2%, compared with that in the same months last year. Clinical studies have found that TCM participation in cancer treatment can improve the quality of life and reinforce the effects of chemotherapy and radiotherapy [8, 9]. In addition, TCM treatment can increase the survival time of cancer patients and reduce overall medical costs [10, 11]. Cancer patients with or without cancer-related treatment are vulnerable populations as they are immunocompromised. We speculate that because TCM is deeply ingrained into Taiwan’s cancer treatment and is expected to improve or boost immune function, the TCM medical needs for cancer patients, therefore, increased even during the COVID-19 pandemic.

The COVID-19 pandemic has spread globally for nearly a year, and there is no sign of stopping. This study found that COVID-19 will significantly affect the utilization rate of TCM, but its long-term impact on medical quality is still unknown. This research could be the basis for future long-term medical effects and policy research.

The Taiwan government has absorbed the 2003 SARS prevention and control experience. The government has adopted stricter epidemic prevention measures, including prohibiting medical professionals from going abroad to ensure enough labor to deal with this pandemic. The Immigration Agency has inputted entry and exit information and contact history records into the National Health Insurance (NHI) PharmaCloud system. All physicians can log in to the system and query information. The government directly distributes medical supplies to each doctor through the medical association. The patient and anyone in contact with the patient must be isolated at home for 14 days. The health department of the local government will monitor body temperature and symptoms of infection. Taiwanese people actively cooperate with government policies, including maintaining social distancing, avoiding public places, wearing masks correctly, and washing hands frequently.

There are several research limitations in this article. First, Taiwan’s medical policies and general medical treatment patterns have undergone significant changes from 2003 to 2020. Directly comparing the TCM of 2 years may lead to research bias. Therefore, we use the number of outpatients in the year before the coronavirus epidemic as a benchmark to calculate the change in the number of patients to reduce the interference caused by different time points. Second, we use different research samples to compare the TCM usage rates during two large-scale coronavirus outbreaks. In 2003 we used a national sample population sample, and in 2020 we used the Taipei City Hospital survey. However, there is no significant statistical difference in the distribution of TCM treatment department and treatment frequency. Third, our research focuses on the comparison of the number of visits by TCM. Due to the Institutional Review Board’s restriction, we couldn’t obtain detailed demographics data such as area, income, age, and educational level. According to the Taipei City Government’s demographic data, the death rate in Taipei City is approximately six deaths per 1000 people (6.33 in 2015, 6.66 in 2016, 6.48 in 2017, 6.69 in 2018, and 6.78 in 2019) [12]. Since the statistics of mortality in Taiwan in 2020 have not yet been announced, we cannot assess the impact of mortality on Chinese medicine utilization. However, because COVID-19 has not spread widely in Taiwan, only 776 people were infected, and seven people died in December 2020. We estimate that the COVID-19 mortality rate will not significantly change the utilization rate of TCM.

## Conclusion

Preemptive preparedness to fight COVID-19 is paramount. The COVID-19 pandemic and restrictive health policies decreased the TCM utilization rate, especially for mobile healthcare in rural areas. We therefore conclude that there should be adequate fund allocation for the healthcare system and that TCM medical workforce for cancer patients should be provided to maintain medical access and impartiality in terms of resource allocation in rural areas, especially during the pandemic.

## Data Availability

The datasets generated or analyzed during the current study are not publicly available due privacy policy but are available from the Medical Research Center of Taipei City Hospital on reasonable request.

## Abbreviations

TCM: Traditional Chinese medicine
COVID-19: Coronavirus disease 2019
SARS: Severe Acute Respiratory Syndrome
